# Development of the Health Literacy of Caregivers Scale - Cancer (HLCS-C): item generation and content validity testing

**DOI:** 10.1186/s12875-014-0202-9

**Published:** 2014-12-10

**Authors:** Eva YN Yuen, Tess Knight, Sarity Dodson, Lina Ricciardelli, Susan Burney, Patricia M Livingston

**Affiliations:** School of Psychology, Deakin University, 221 Burwood Highway, Burwood, VIC 3125 Australia; Public Health Innovation, Deakin University, Melbourne, Australia; School of Psychological Sciences, Monash University, Melbourne, Australia; Faculty of Health, Deakin University, Melbourne, Australia

**Keywords:** Cancer, Caregivers, Health literacy, Information needs, Questionnaire development

## Abstract

**Background:**

Health literacy refers to an individual’s ability to engage with health information and services. Cancer caregivers play a vital role in the care of people with cancer, and their capacity to find, understand, appraise and use health information and services influences how effectively they are able to undertake this role. The aim of this study was to develop an instrument to measure health literacy of cancer caregivers.

**Method:**

Content areas for the new instrument were identified from a conceptual model of cancer caregiver health literacy. Item content was guided by statements provided by key stakeholders during consultation activities and selected to be representative across the range of cancer caregiver experiences. Content validity of items was assessed through expert review (*n* = 7) and cognitive interviews with caregivers (*n* = 16).

**Results:**

An initial pool of 82 items was generated across 10 domains. Two categories of response options were developed for these items: agreement with statements, and difficulty undertaking presented tasks. Expert review revealed that the majority of items were relevant and clear (Content Validity Index > 0.78). Cognitive interviews with caregivers suggested that all except three items were well understood.

**Conclusion:**

A resultant 88 item questionnaire was developed to assess cancer caregiver health literacy. Further work is required to assess the construct validity and reliability of the new measure, and to remove poorly performing and redundant items, which will result in a shorter, final measure. The new measure has the potential to inform the development and evaluation of interventions and the improvement of health service delivery to cancer caregivers.

## Background

A diagnosis of cancer impacts not only the person diagnosed, but also their family members and friends. These social supports are often called upon to provide informal care and assistance managing the disease [1] and to provide practical, emotional and physical support [2]. Individuals who provide informal care and support, often referred as caregivers [3], also play a significant role in health-related decision-making [4], are involved in communications with healthcare providers [5], and assist with sourcing and interpreting health information [6]. These caregiving responsibilities are often undertaken unexpectedly, and caregivers are often provided limited information and support [1]. Recognition of the challenges of the caregiving role has led to development of interventions designed to meet the informational, practical, and psychosocial needs of caregivers [7–9]. Although information provision is included in the majority of these interventions [10], few studies have examined improvements in the level of caregivers’ knowledge and skills [7,10]. This may, in part, be due to the lack of measurement tools that assess caregiver knowledge and skills [2].

Consistent with broad definitions of health literacy [11–13], caregiver health literacy is defined here as the personal characteristics and social resources needed for caregivers to access, understand, appraise and use information and services to participate in decisions relating to the health and care of the care recipient. This includes the capacity to communicate, assert and enact these decisions. Whilst evidence suggests an association between poor health literacy and poorer health outcomes [14], worse physical functioning and reduced quality of life [14–18], little is understood about the relationship between caregiver health literacy and the health outcomes of care recipients.

To accurately identify the health literacy needs of cancer caregivers, and understand the impact of caregiver health literacy on care recipient health outcomes, it is essential to measure the construct effectively. Previous studies of caregiver health literacy [19–22] have used measures that assess a subset of health literacy constructs. Measures such as the Test of Functional Health Literacy in Adults (TOFHLA [23]) or its short form [24], the Rapid Estimate of Adult Literacy in Medicine [25], and the Newest Vital Sign [26] assess an individual’s reading, numeracy, and comprehension skills in relation to healthcare. Reviews of health literacy measurement instruments increasingly call for the development of tools that capture the full range of health literacy constructs [27–29], such as critical thinking, interaction and communication, and confidence [11].

In response to this gap in the literature, health literacy measurement tools are now emerging that capture the multidimensional nature of health literacy [30,31]. However, these tools are grounded in the perspectives of the potential care recipient, and have limited utility for the identification of the needs of caregivers. Similarly, caregiver health literacy measures designed to assess health literacy of parents of infants [32,33] cover domains not relevant to the role of caregiving for an adult recipient.

**Figure 1:**
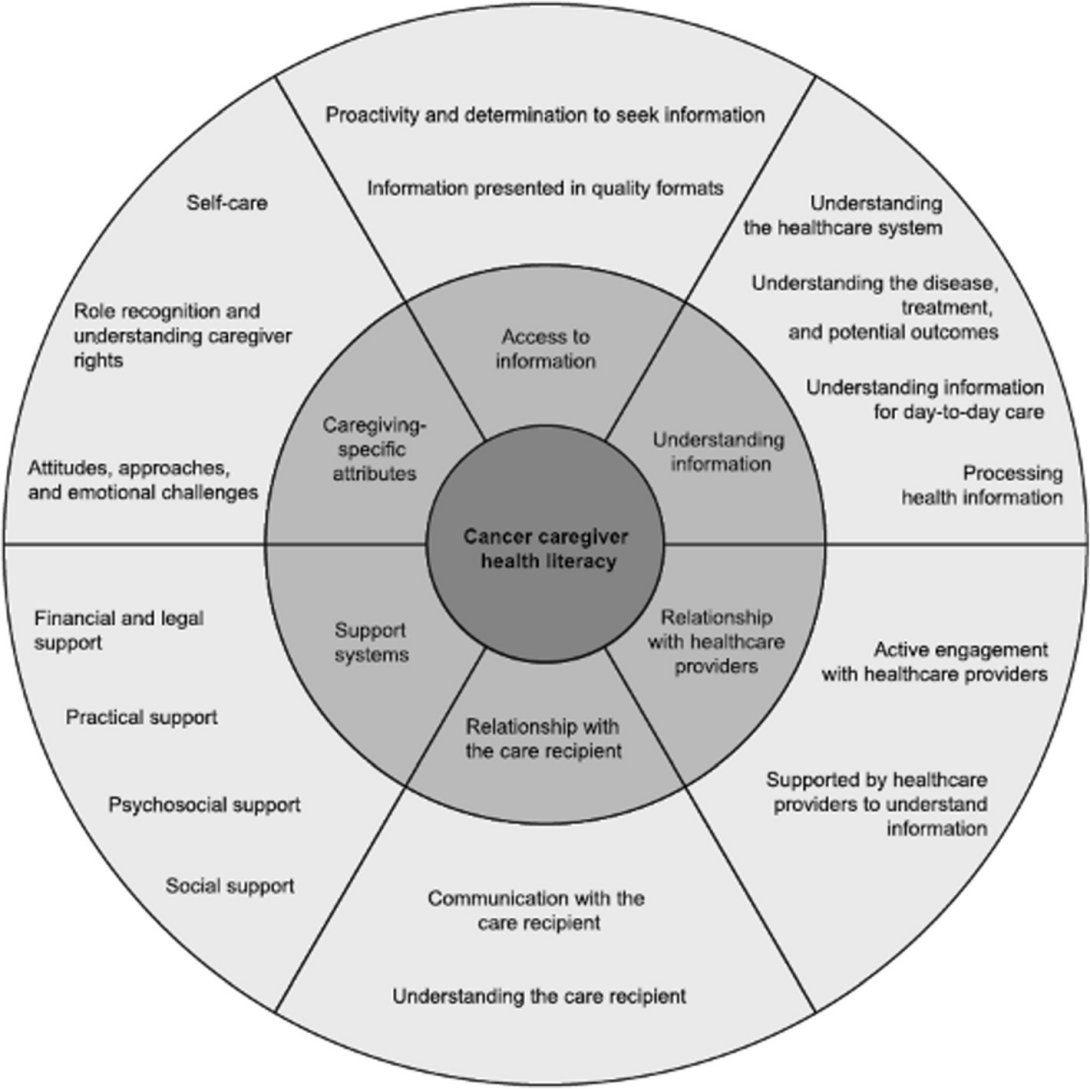
**Conceptual model of cancer caregiver health literacy (Yuen et al., in press).**

The aim of the current study was to develop a measure of health literacy specifically for caregivers of people with cancer. Best practice guidelines for questionnaire development require a detailed conceptual basis to guide development [34,35]. The conceptual model of caregiver health literacy developed by the authors (Yuen, Dodson, Batterham, Knight, Chirgwin, & Livingston, in press) was used as the basis for the development of the Health Literacy of Caregivers Scale - Cancer (HLCS-C). The model, as shown in Figure 1, proposes six major themes and 17 sub-themes associated with caregiver health literacy.

## Methods

**Figure 2:**
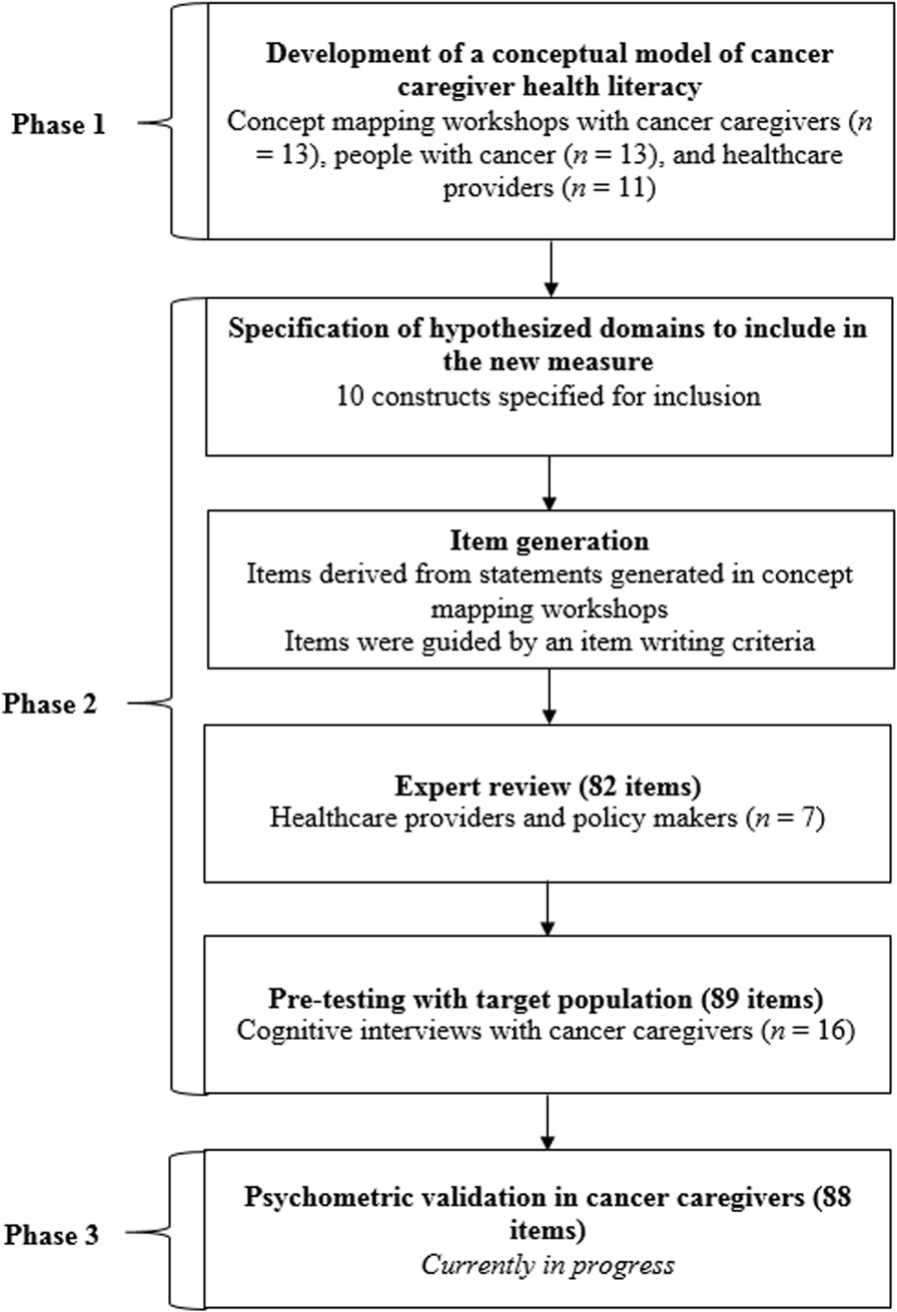
**Steps undertaken to develop items for the new measure of cancer caregiver health literacy.**

A validity-driven approach [36] was employed in the development of the HLCS-C. The steps undertaken are outlined in Figure 2. The study was approved by the Eastern Health Human Research Ethics Committee (E41-1011) and Deakin University Human Research and Ethics Committee (2011–115), in Melbourne, Australia.

### Content area specification

The content areas for inclusion in the questionnaire were drawn directly from 17 sub-themes in the conceptual model of cancer caregiver health literacy (see Figure 1). The following considerations were used to determine whether (and how) themes should be represented in the questionnaire: 1) the questionnaire should capture the experiences of caregivers caring for recipients with a wide range of cancer types, stages, treatments, and potential outcomes; 2) the questionnaire should capture the experience of caregivers providing differing forms and levels of support; 3) the questionnaire should be consistent with the broad definition of caregiver health literacy, and encompass factors associated with *accessing*, *understanding*, *appraising* and *using* health information to promote and maintain the health of the care recipient; 4) the questionnaire should be presented as a list of items/statements accompanied by an appropriate set of response options; and 5) the questionnaire should contain the fewest number of domains as possible to reduce length and administration burden.

Another consideration when identifying content areas for inclusion was whether representative statements generated by participants during consultation activities captured caregiver experiences or whether they captured broader contextual factors that influenced caregiver health literacy. In addition, content areas were examined to determine whether statements representative of a sub-theme could be combined to form a scale; previous scale development studies that used similar processes to derive a conceptual model, have found that although statements within some sub-themes were conceptually related, could not be summed to form a scale score, and required deletion on psychometric grounds [30]. Further, to assist cross-referencing of the new measure against other measurement tools that assess related constructs, the included content areas were also aligned with a recently developed taxonomy that identified 12 dimensions of health literacy [11]: literacy; interaction; comprehension; numeracy; information seeking; application/function; decision making/critical thinking; evaluation; responsibility; confidence; navigation; and maintaining and promoting health (Table 1).

**Table 1:** **Specification of the ten scales hypothesized to define cancer caregiver health literacy, reasons for exclusion of content areas, and example items for each scale**

**Content area identified in conceptual model**	**Draft scale included in pre-testing/Reason for exclusion**	**Example item**	Health literacy dimension*	**Number of items included in each scale**			**Response options**
				**Expert review**	**Cognitive interviews**	**Psychometric assessment**	
1. Proactivity and determination to seek information	1. Proactivity and determination to seek information	I keep looking until I get all the information that I need	Information seeking	9	9	8	Agree/disagree
2. Information presented in quality formats	*Subsumed into “Adequate information about cancer and cancer management” to minimize questionnaire length*		-	-	-	-	-
3. Understanding the healthcare system	2. Understanding the healthcare system	I understand what healthcare services the person I care for is entitled to	Comprehension	7	9	9	Agree/disagree
4. Understanding the disease, treatment, and potential outcomes	3. Adequate information about cancer and cancer management	I have all the information I need to help look after the health of the person I care for	Comprehension	8	8	8	Agree/disagree
5. Information for day-to-day care	*Subsumed into “Adequate information about cancer and cancer management” to ensure relevance of items to all caregivers*		-	-	-	-	-
6. Processing health information	4. Processing health information	[Please indicate how easy or difficult the following tasks are for you to do now:] Compare information about cancer from different sources	Critical thinking/evaluation	8	9	9	Difficulty
7. Active engagement with healthcare providers	5. Active engagement with healthcare providers	[Please indicate how easy or difficult the following tasks are for you to do now:] Ask a healthcare provider to explain things to me	Interaction	7	8	8	Difficulty
8. Supported by healthcare providers to understand information	6. Supported by healthcare providers to understand information	At least one healthcare provider has helped me understand information about cancer	Support networks**	8	10	10	Agree/disagree
9. Communication with the care recipient	7. Communication with the care recipient	I have honest talks with the person I care for about how the cancer may impact on the future	Interaction	8	8	8	Agree/disagree
10. Understanding the care recipient	8. Understanding the care recipient	I know how much help to give the person I care for	Comprehension	9	9	9	Agree/disagree
11. Financial and legal support	*Considered a broader contextual factor related to availability of support from Government services*			-	-	-	-
12. Practical support	*Considered a broader contextual factor related to availability of support from community services*			-	-	-	-
13. Psychosocial support	*Subsumed into “Understanding the healthcare system” to ensure relevance of items to all caregivers*			-	-	-	-
14. Social support	9. Social support	I have at least one person who understands and supports me	Support networks**	9	7	7	Agree/disagree
15. Self-care	10. Self-care	I regularly take time away from caring	Responsibility	11	12	12	Agree/disagree
16. Role recognition and understanding caregiver rights	*Statements within the sub-theme although conceptually related were considered unable to be additively combined, thus were excluded from the scale*			-	-	-	-
17. Attitudes, approaches, and emotional challenges	*Statements within the sub-theme although conceptually related were considered unable to be additively combined, thus were excluded from the scale*			-	-	-	-
	Total items in scale			84	89	88	

*Adapted from health literacy dimensions identified by Sorensen et al. [11].

**Additional dimension included by authors, not identified in taxonomy.

### Generation of items and response scale

Statements and words provided by participants during consultation activities associated with the development of the cancer caregiver health literacy conceptual model (see Table 1) were used as the starting point for questionnaire items [37] to maximize content validity. For each content area, item selection and refinement was guided by two vignettes developed to describe an individual with a high degree of capacity in that area, and the other with low levels [30]. Where the proposed content areas for the new measure were similar to domains included in the Health Literacy Questionnaire (HLQ; [30]), a validated measure of health literacy derived using similar approaches, the HLQ items were used as the basis and revised to accommodate the caregiver audience. Response scales for each content area were developed to match the nature of the associated items and vignettes. Refinements to how content areas and vignettes were framed were undertaken to ensure consistency in response scales across the content areas of the proposed questionnaire. Items were also examined against a structured item development criteria [38] (see Table 2). Readability of items was assessed using Flesch Reading Ease [39] and Flesch-Kincaid Grade Level [40] formulas available through Microsoft Word.

**Table 2:** **Structured item development criteria used to assess quality of items**

**#**	**Criteria to assess item quality**	**Possible outcome**	**Acceptable outcome to retain item**
1	How difficult is the item for respondents endorse the maximum score	Very difficult; Moderately difficult; Easy	All three possible outcomes. Author sought to develop constructs that contained items with a range of difficulty
2	How comprehensible is the item for caregivers with high and low literacy	Comprehensible; Contains words that may be difficult for caregivers to understand	Comprehensible
3	How relevant is the item for respondents of different ages	Relevant to caregivers ages 18 years and above; Not relevant to specific age groups (e.g., elderly)	Relevant to caregivers ages 18 years and above
4	How pertinent is the item to the associated content area	Critical/Core; Important; Relevant	Critical/Core; Important
5	How relevant is the item to all members of the target population (i.e., caregivers of adults with cancer)	Relevant to caregivers across the cancer spectrum; Specific to caregiving experiences along cancer spectrum	Relevant to caregivers across cancer spectrum
6	How independent is the item to other items	Moderately independent; Too closely related to one or more items	Moderately independent
7	How well does the item fit with other items in the construct	Fits well; Different content or meaning to other items in construct	Fits well
8	Does the item capture a single idea (or two closely related ideas)	Yes; No	Yes
9	How minimal are the information processing demands	One or two processing demands; More than one or two processing demands	One or two processing demands
10	Does the item stem correspond to the response scale	Yes; No	Yes

Item difficulty was included in the item development criteria to ensure that the final items formed a scale that could distinguish between low, moderate, and high levels of health literacy (i.e., scale sensitivity). The revised Bloom’s taxonomy, which includes two dimensions (knowledge and cognitive process; [41,42]) was used to guide the selection of set of items for each content area to ensure they captured a range of difficulty. The first Bloom dimension describes levels of knowledge acquired (factual, conceptual, procedural, or metacognitive) whilst the second dimension describes cognitive processes that occur during learning (remembering, understanding, applying, analyzing, evaluating, and creating; [41,42]). It is posited that items that address higher level cognitive tasks (e.g., decision-making) would elicit fewer maximum ratings compared to items that addressed lower level cognitive tasks (e.g., access to information). The taxonomy has been previously used to guide the development of health literacy measures [30,43,44].

### Expert review

Expert review of items was undertaken to establish the content validity of the proposed items [45]. In a judgment-quantification process [46], items within each proposed scale were assessed by seven experts for relevance and clarity. Participants included two oncologists, a general practitioner, an oncology social worker, a general medical nurse, a health researcher, a policy advisor for a state-wide caregiver organization, and a retired executive member of a cancer information and support service. The content validity of the tool as a representation of its intended purpose was also qualitatively assessed. Experts were identified and recruited from the research team’s existing professional networks. Between 5 and 10 experts have been suggested as a number sufficient for establishing content validity using expert review [46].

Experts were asked to assess each item for relevance and clarity using a 3-point scale (“low, moderate, high” and “unclear, neutral, clear” respectively). To determine content validity, expert ratings for relevance and clarity were quantified using the Content Validity Index (CVI) calculated as the percentage of experts who indicated 2 or 3 on the scale. It has been recommended that when six or more experts have evaluated the instrument, items with a CVI less than 0.78 should be considered for revision or deletion [46].

Experts were also asked to consider all items within individual scales and respond to two open-ended questions, “Do you suggest including any other ideas to represent the scale”, and “Do you suggest changing any words for any of the above items”. Experts were also asked to provide feedback on whether any major concepts or ideas were omitted in the questionnaire and to make suggestions on how to improve the instrument. To guide the revision of items, responses to the open-ended questions were synthesized and reviewed.

### Cognitive interviews

Cognitive interviews are frequently used in questionnaire development to determine whether respondents interpret and respond to items in the way the researchers intended [47]. The think-aloud approach [47,48] was the predominant method used in the current study. A convenience sample of participants was recruited from a not-for-profit government funded caregiver organization. Ninety-nine caregivers who identified themselves as providing care to a family member or friend with cancer were invited to participate. Nineteen caregivers (19%) who returned the questionnaire were then contacted via telephone about taking part in a telephone interview. Three respondents completed the questionnaire; however, they declined to participate in the cognitive interview because of personal circumstances. Of the 16 caregivers who participated, the majority were female (94%), and ranged in age between 42 and 80 years (*Mdn =* 61.5; see Table 3).

**Table 3:** **Demographic characteristics of caregivers who participated in cognitive interviews**

**Demographic characteristics**	**Caregivers (** ***n*** **= 16)**	
	***n***	**%**
Gender – Female	15	94%
Age (years)		
≤ 65	11	69%
≥ 66	5	31%
Care recipient cancer type		
Hematological	12	75%
Solid	4	25%
Length of time as a caregiver		
1 to 2 years	3	19%
2 to 4 years	6	37.5%
More than 5 years	6	37.5%
Unspecified	1	6%
Education		
Completed some or all high school	7	44%
Completed some or all of University	9	56%
Speaks English at home	16	100%
Caregiver relationship to care recipient		
Spouse	9	56%
Parent, sibling, or child	5	31%
Friend	2	13%

To minimize respondent burden, a sampling scheme was applied to allow each participant to be interviewed on items from approximately 6, rather than all 10, constructs in the questionnaire. Participants were randomly assigned an item set that included items from complete constructs. Using this method, each item in the questionnaire was reviewed by at least 8 participants (range = 8 – 11; *Mdn* = 9). Although participants did not complete the full set of items, the sampling scheme was sufficient as the purpose of the cognitive interviews was to test the items across a range of individuals to inform decision making [47].

Responses from the cognitive interviews were analyzed using a systematic evaluation of participant responses for each item [49]. Each item was assessed using three criteria: whether the participant interpreted the question as the researchers had intended; whether the item was applicable to the participant; and whether the participant found it difficult to respond to the item. In cases where responses had problems with an item, common themes and issues were noted.

## Results

### Selection of content areas

Inspection of the 17 sub-themes outlined in the cancer caregiver health literacy model against the considerations for inclusion of content areas led to the identification of 10 constructs for the new questionnaire (see Table 1). Several sub-themes were subsumed under the encompassing scale titles: Adequate information about cancer and cancer management, and Understanding the healthcare system. Two sub-themes were considered broader contextual factors that influenced caregiver health literacy, and thus were excluded from the questionnaire. For example, statements in the Financial and Legal Support sub-theme related to availability of support from Government services, which was considered a broader contextual factor that influenced a caregiver’s capacity to effectively engage with the caregiving role. Two additional sub-themes were excluded because their representative statements, although conceptually related, were considered unable to be summed to form a scale.

### Item generation and response options

Eighty-two items were developed for expert review, with 7 to 12 items for each construct (see Table 1). An item pool 50% larger than that intended for the final scale was drafted to enable identification of items with adequate internal consistency as determined through psychometric analyses (Phase 3; see Figure 2) [45]. For eight content areas, an ‘agree/disagree’ Likert scale was suitable. For the remaining two content areas (Processing health information, and Active engagement with healthcare providers) a ‘cannot do/very easy’ Likert scale was more suitable. Readability analysis of the items showed a Flesch-Kincaid reading level of grade 6.7, with a Flesch reading ease of 80.6 (out of a possible 100, with higher scores indicating greater ease).

### Expert review

The range of content validity indices for relevance and clarity for the ten scales as assessed by 7 experts are provided in Table 4. Although 8 experts responded, one participant provided general comments about including additional content areas rather than assessing all individual items, thus, was excluded from the content validity analysis. The participant’s comments were considered when determining the inclusion of additional content areas. Items were considered relevant by experts (CVI > 0.78) for all but one item related to processing health information (#70, “*Find out if the health information that I have received is suitable for the person I am caring for*”). Item #70 was considered invalid both for relevance and clarity (CVI < 0.78), and thus was revised (see Table 5) after considering expert comments, and reviewing participant statements generated during concept mapping workshops.

**Table 4:** **Range of CVI scores for relevance and clarity for ten hypothesized scales of cancer caregiver health literacy**

**#**	**Construct**	**Relevance**		**Clarity**	
		**CVI range**	**Items with CVI <0.78**	**CVI range**	**Items with CVI <0.78**
1	Proactivity and determination to seek information	0.86 – 1.00	-	0.86 – 1.00	-
2	Understanding the healthcare system	1.00	-	0.86 – 1.00	-
3	Adequate information about cancer and cancer management	1.00	-	0.71 – 1.00	#42
4	Processing health information	0.71 – 1.00	#70	0.43 – 1.00	#70
5	Supported by healthcare providers to understand information	0.86 – 1.00	-	0.86 – 1.00	-
6	Active engagement with healthcare providers	1.00	-	0.86 – 1.00	-
7	Communication with the care recipient	0.86 – 1.00	-	0.71 – 1.00	#21, #37
8	Understanding the care recipient	0.86 – 1.00	-	0.71 – 1.00	#6
9	Social support	1.00	-	1.00	-
10	Self-care	0.86 – 1.00	-	0.86 – 1.00	-

**Table 5:** **Seven revised items in response to content validity index scores for relevance and clarity, and comments from experts**

**Construct**	**Item #**	**Initial item**	**Relevance**	**Clarity**	**Comments from experts**	**Action**	**Revised item**
			CVI	CVI			
Understanding the healthcare system	81	I know which healthcare providers look after the health of the person I care for	1.00	1.00	Almost identical to another item in the scale	Deleted	-
Adequate information about cancer and cancer management	42	I am sure I have all the information I need to help manage the health of the person I care for	1.00	0.71	Item #42 and #64 are similar	Revised	I have enough information to look after the health of the person I care for
Processing health information	70	Find out if the health information that I have received is suitable for the person I am caring for	0.71	0.43	Implies that health information e.g. by healthcare providers is not suitable	Revised	Find out if the health information that I have found from various resources, is suitable for the person I am caring for
Communication with the care recipient	21	The person I care for tells me how they are, in order for me to help	1.00	0.71	Items #21 and 29 are similar, but also very general in description	Revised	The person I care for tells me about their health, in order for me to help
	37	I talk honestly about the cancer with the person that I care for	1.00	0.71	Item seems general	Revised	I have honest talks about the cancer with the person I care for
Understanding the care recipient	6	I understand how much information about the cancer, the person I am caring for needs to know	0.86	0.71	There is a difference between ‘needs’ and ‘wants’	Revised	I understand how much information about the cancer, the person I am caring for wants to know

Using the content validity equation, five items although deemed relevant by experts, were considered to lack clarity (CVI < 0.78; see Table 4). These five items were revised (see Table 5). An additional item (#81, “I know which healthcare providers look after the health of the person I care for”), although deemed relevant and clear, was deleted in response to expert comments about its similarity to another item in the scale.

Twelve items, although demonstrated adequate relevance and clarity (CVI > 0.78), underwent minor revisions in response to suggested improvements from experts (See Table 6). Item #12 (“I have strong support from at least one friend” was combined with item #66 (“I have strong support from at least one family member) following feedback about the similarity of items, and suggestions from experts to merge the two items.

**Table 6:** **Revised items following expert suggestions for revision**

**Construct**	**Item #**	**Initial item**	**Revised item**	**Comments from experts**
Supported by healthcare providers to understand information	50	Healthcare providers have helped us compare information about treatments	Healthcare providers have helped me compare information about treatments	Consider using ‘me’ rather than ‘us’ to avoid confusion
	57	Healthcare providers have helped me understand the potential side effects of treatments	Healthcare providers have helped us understand the potential side effects of treatments	Consider using ‘me’ rather than ‘us’ to avoid confusion
Communication with the care recipient	58	After appointments, I discuss the information given by doctors with the person I care for	After appointments, I discuss the information given by healthcare providers with the person I care for	Referring to doctors or all healthcare providers
Understanding the care recipient	14	I understand when to let the person I am caring for do things by themselves in their own time	I understand when to let the person I am caring for do things for themselves in their own time	Perhaps “for themselves” better captures the concept
	30	I know which everyday activities the person I care for would like to be involved in	I know which everyday activities the person I care for would like to do	Changing “involved in” might improve clarity
		I know which everyday activities the person I care for can participate in	I know which everyday activities the person I care for can do	Changing “participate in’ to “can do” might improve clarity
Social support	4	There is at least one person who understands and supports me	I have at least one person who understands and supports me	Consider changing the stem to follow other items
	12	I have strong support from at least one family member	I have strong support from at least one family member or friend	Is it necessary to differentiate between family member and friend
	66	I have strong support from at least one friend	*Not applicable: Subsumed into above item*	As above
	20	I get plenty of chances to talk to other people who are caring for someone with cancer	I get enough chances to talk to other people who are caring for someone with cancer	Might not need ‘plenty’
	44	I have family or friends who can attend medical appointments with us	I have at least one family member or friend who can attend medical appointments with us	Identifying one person would be adequate

Nine new items were included in the questionnaire in response to comments from experts (see Table 7). Revision of the item pool resulted in 89 items for testing through cognitive interviews. Experts identified three main areas that were missing from the questionnaire: understanding of healthcare services, palliative care, and sexuality issues. However, only the concept of understanding of healthcare services was captured in newly generated items.

**Table 7:** **New items following expert review and reasons for inclusion**

**Construct**	**Comments from experts**	**New item**
Understanding the healthcare system	(General comment) It is important for caregivers to understand what services and supports are available for the caregiver and care recipient	I know what healthcare services are available to help the person I care for
Adequate information about cancer and cancer management	Suggest including additional items about managing side effects, and caregivers’ having enough information to support the care recipient	I know which side-effects require immediate medical attention
		I know the routine things the person I care for needs to do to look after their own health
		I know what healthcare services are available to help me
Processing health information	Suggest including additional questions that explore caregiver’s capacity to identify relevant information	[How easy or difficult is it for you to…] Work out which sources have information that is relevant for the person I care for
Supported by healthcare providers to understand information	(General comment) It is important for caregivers to understand what services and supports are available for the caregiver and care recipient	Healthcare providers have helped me understand services available for the person I care for
		Healthcare providers have helped me understand services available to support me
Active engagement with healthcare providers	(General comment) It is important for caregivers to understand what services and supports are available for the caregiver and care recipient	Ask a healthcare provider to explain what healthcare services are available to help me provide care
Self-care	Suggest including a question about physical activity or exercise	Despite other things in my life, I make sure I regularly exercise

### Cognitive interviews

Overall, participants interpreted and responded to the majority of the questionnaire items in ways intended. However, three items (#18, #74 and #1) emerged as having common issues. For item #18 (“I have all the information I need to help make decisions about treatments”) two participants reported that they did not help make decisions about treatments, thus the item was not personally relevant to them (e.g. “*I’m not a doctor and I wouldn’t know of other treatments, so I trusted what doctors told me*” [Participant 1]). For item #74 (”Find out if health information from various resources is suitable for the person I am caring for”) participants interpreted the word ‘resource’ as being internet-specific (e.g. “*Yeah I think it is, you just borrow the kids internet and have a look*” [Participant #11]). Further, for item #1 (“I spend a lot of time looking for information about the cancer”) two caregivers reported that although they spent time looking for information when their care recipient was first diagnosed with cancer, it was no longer relevant after many years of providing care (e.g. “*My husband has had cancer now for years. At the beginning I spent a lot of time researching but now only when you feel up to it*” [Participant #10]).

## Discussion

The current study describes item generation and content validity testing of a new questionnaire to assess the self-reported health literacy of caregivers of people with cancer, the Health Literacy of Caregivers Scale–Cancer (HLCS-C). As a result of the expert review and cognitive interviews, the HLCS-C now contains 88 items across 10 scales: proactivity and determination to seek information; adequate information about cancer and cancer management; supported by healthcare providers to understand information; social support; communication with the care recipient; understanding the care recipient; self-care; understanding the healthcare system; processing health information; and active engagement with healthcare providers.

The scales included in the HLCS-C covered a broad range of themes that assessed individual, interpersonal as well as healthcare provider and healthcare system factors that may be relevant to caregiver health literacy. Many of these themes are currently not included in widely-used measures of health literacy. For example, some scales in the HLCS-C assess an individual’s comprehension (e.g., Adequate information about cancer and cancer management, and Understanding the healthcare system), or their critical thinking skills (e.g., Processing health information), while other scales assess a caregiver’s interpersonal relationship with the care recipient (e.g., Communication with the care recipient, and Understanding the care recipient). Caregivers’ capacity to effectively engage with healthcare providers was also included (Active engagement with healthcare providers). Further extending dimensions of health literacy measures, the HLCS-C assesses external influences on an individual’s health literacy. Similar to the Health Literacy Questionnaire [30], the HLCS-C contains a scale that assesses the caregivers’ perspectives of healthcare provider provision of services and information in ways that enable them to adequately navigate the caregiving role and the healthcare system (e.g., Supported by healthcare providers to understand information). Unlike the existing unidimensional measures of health literacy, the multidimensional nature of the HLCS-C allows identification of specific strengths and difficulties and therefore the identification of opportunities to improve caregiver health literacy and the health literacy responsiveness of the healthcare system.

As part of the expert review, experts suggested including items related to sexuality issues. However, the authors made the decision to not include items related to sexuality issues as this topic was not identified by stakeholders during the concept mapping workshops. Concept mapping workshop participants included caregivers providing care for, and people with cancer, across a range of cancer types and stages. It is possible that issues of sexuality were not their primary concern when identifying health literacy needs. Further, it is possible that given the workshop setting, participants may have felt uncomfortable discussing the topic of sexuality. Moreover, studies have shown that caregivers of people with gender-specific cancers (e.g., breast or prostate) were more likely to report additional information needs related to sexual and physical intimacy [50]. Further revisions of the questionnaire could consider sub-sets of items relevant to specific cancer types.

Similarly, experts commented on the inclusion of items related to palliative care. However, the questionnaire was designed for use with caregivers across the cancer trajectory. Thus, the authors considered that items related to palliative care would not be relevant to all cancer caregivers. Future revisions of the questionnaire could consider items that are specific to caregivers providing care to people with advanced stage cancer.

To address the three items identified as having common issues following cognitive interviews, the decision was made to revise two items and delete one item. To ensure included items were relevant to all cancer caregivers, item #18 was revised to “I have enough information to understand the potential side effects of cancer treatment”, which still captured the concept of adequate cancer information. To improve clarity for item #74, the word ‘resources’ was replaced with ‘places’, as participants frequently used this word during cognitive interviews to describe sources of information. Further, as item #1 was not relevant for all caregivers across the caregiving trajectory, the item was deleted. Cognitive testing of the revised items is suggested to ensure items are understood as intended.

Two of the 16 participants responded with ‘disagree/very difficult’ on five items, which suggested that they had difficulty, or were unable to complete that task. However, during the cognitive interviews, it was revealed that these participants had provided care to someone who had deliberately avoided conventional cancer treatments for exclusive use of complementary and alternative therapies to manage the cancer. Thus, in responding to specific items, these participants were not conveying difficulty or inability to complete the task; rather their intention was to convey that the item was ‘not applicable’ to their circumstance. Item writing was guided by statements generated by participants during concept mapping workshops who were recipients of, or caregivers of people who received, conventional cancer treatments. It is therefore recommended that future studies be conducted with caregivers of people who solely receive complementary and alternative therapies to manage their cancer to ensure a sub-set of items that address the health literacy needs of this caregiver population.

Limitations of the study included the low response rate for expert reviews (29%). Although low response rates may potentially affect generalizability of the results, the sample size for the expert review analysis was in line with recommendations [51]. Participation rate was also low for the cognitive interviews (19%); however, between 8 to 11 interviews were conducted for each item, which met the recommended sample size of 5 to 15 participants to identify problems with items [47]. Further, participants for cognitive interviews were predominantly female (94%), which limits generalizability of the findings. Further, reporting error may occur due to the self-report nature of the questionnaire, in which respondents may report differently depending on their social experiences [52].

## Conclusion

Using systematic grounded approaches, a new measure of cancer caregiver health literacy is being developed that contains 10 key constructs hypothesized to represent a caregiver’s capacity to find, understand, appraise, and use health information to provide optimal care. The next step in the development of this measure is to assess the reliability and validate the questionnaire in a large sample of Australian cancer caregivers, and reduce the number of items it contains.

### Practice implications

The current study represents the first attempt to establish an instrument to measure the health literacy of caregivers of people with cancer. Assessment and understanding of the health literacy needs of caregivers has the potential to enable the evaluation and development of interventions designed to improve caregiver knowledge and skills.

### Consent

Written informed consent was obtained from caregivers for the publication of this report.

## Abbreviations

CVI: Content Validity Index
HLCS-C: Health Literacy of Caregivers Scale-Cancer
HLQ: Health Literacy Questionnaire
TOFHLA: Test of Functional Health Literacy in Adults

## References

[1] van Ryn M, Sanders S, Kahn K, van Houtven C, Griffin JM, Martin M, Atienza AA, Phelan S, Finstad D, Rowland J (2011). Objective burden, resources, and other stressors among informal cancer caregivers: a hidden quality issue?. Psycho-Oncology.

[2] Given BA, Given CW, Sherwood PR (2012). Family and caregiver needs over the course of the cancer trajectory. J Support Oncol.

[3] Reinhard SC, Given B, Petlick NH, Bemis A, R.G. H (2008). Supporting Family Caregivers in Providing Care. Patient safety and quality: An evidence-based handbook for nurses. Volume 1.

[4] Hubbard G, Illingworth N, Rowa-Dewar N, Forbat L, Kearney N (2010). Treatment decision-making in cancer care: the role of the carer. J Clin Nurs.

[5] Laidsaar-Powell RC, Butow PN, Bu S, Charles C, Gafni A, Lam WWT, Jansen J, McCaffery KJ, Shepherd HL, Tattersall MHN (2013). Physician–patient–companion communication and decision-making: a systematic review of triadic medical consultations. Patient Educ Couns.

[6] Bevan JL, Pecchioni LL (2008). Understanding the impact of family caregiver cancer literacy on patient health outcomes. Patient Educ Couns.

[7] Applebaum AJ, Breitbart WS (2013). Care for the cancer caregiver: a systematic review. Palliat Support Care.

[8] Badr H, Krebs P (2013). A systematic review and meta‐analysis of psychosocial interventions for couples coping with cancer. Psycho-Oncology.

[9] Hudson PL, Remedios C, Thomas K (2010). A systematic review of psychosocial interventions for family carers of palliative care patients. BMC Palliat Care.

[10] Northouse LL, Katapodi MC, Song L, Zhang L, Mood DW (2010). Interventions with family caregivers of cancer patients: meta‐analysis of randomized trials. CA Cancer J Clin.

[11] Sorensen K, Broucke SV, Fullam J, Doyle G, Pelikan J, Slonska Z, Brand H (2012). Health literacy and public health: a systematic review and integration of definitions and models. BMC Public Health.

[12] World Health Organization (1998). Health Promotion Glossary.

[13] **Ophelia Toolkit: a step-by-step guide for identifying and responding to health literacy needs within local communities. Part A: introduction to health literacy.** [http://www.ophelia.net.au]

[14] Zhang NJ, Terry A, McHorney CA (2014). Impact of health literacy on medication adherence: a systematic review and meta-analysis. Ann Pharmacother.

[15] Apter AJ, Wan F, Reisine S, Bender B, Rand C, Bogen DK, Bennett IM, Bryant-Stephens T, Roy J, Gonzalez R (2013). The association of health literacy with adherence and outcomes in moderate-severe asthma. J Allergy Clin Immunol.

[16] Berkman ND, Sheridan SL, Donahue KE, Halpern DJ, Crotty K (2011). Low health literacy and health outcomes: an updated systematic review. Ann Intern Med.

[17] Kamimura A, Christensen N, Tabler J, Ashby J, Olson LM (2013). Patients utilizing a free clinic: physical and mental health, health literacy, and social support. J Commun Health.

[18] Bostock S, Steptoe A (2012). Association between low functional health literacy and mortality in older adults: longitudinal cohort study. Br Med J.

[19] Garcia CH, Espinoza SE, Lichtenstein M, Hazuda HP (2013). Health literacy associations between Hispanic elderly patients and their caregivers. J Health Commun.

[20] Lindquist LA, Nelia JBS, Tam K, Martin GJ, Baker DW (2011). Inadequate health literacy among paid caregivers of seniors. J Gen Intern Med.

[21] Greenberg D, Dave M, Cagan PW, Ehrlich A (2009). Health literacy in a geriatrics ambulatory practice: an assessment of older adults and their caregivers. Gerontologist.

[22] Greenberg D, Cho K, Wald-Cagan P, Ehrlich A (2008). Health literacy in a geriatric ambulatory practice: an exploratory study of older adults and their caregivers. Gerontologist.

[23] Parker RM, Baker DW, Williams MV, Nurss JR (1995). The test of functional health literacy in adults. J Gen Intern Med.

[24] Baker DW, Williams MV, Parker RM, Gazmararian JA, Nurss J (1999). Development of a brief test to measure functional health literacy. Patient Educ Couns.

[25] Davis TC, Crouch M, Long SW, Jackson RH, Bates P, George RB, Bairnsfather LE (1991). Rapid assessment of literacy levels of adult primary care patients. Fam Med.

[26] Weiss BD, Mays MZ, Martz W, Castro KM, DeWalt DA, Pignone MP, Mockbee J, Hale FA (2005). Quick assessment of literacy in primary care: the Newest Vital Sign. Ann Fam Med.

[27] Haun JN, Valerio MA, McCormack LA, Sørensen K, Paasche-Orlow MK (2014). Health literacy measurement: an inventory and descriptive summary of 51 instruments. J Health Commun.

[28] Jordan JE, Osborne RH, Buchbinder R (2010). Critical appraisal of health literacy indices revealed variable underlying constructs, narrow content and psychometric weaknesses. J Clin Epidemiol.

[29] Nielsen-Bohlman L, Panzer AM, Kindig DA (2004). Health Literacy: A Prescription To End Confusion.

[30] Osborne RH, Batterham RW, Elsworth GR, Hawkins M, Buchbinder R (2013). The grounded psychometric development and initial validation of the Health Literacy Questionnaire (HLQ). BMC Public Health.

[31] Jordan JE, Buchbinder R, Briggs AM, Elsworth GR, Busija L, Batterham R, Osborne RH (2013). The Health Literacy Management Scale (HeLMS): a measure of an individual’s capacity to seek, understand and use health information within the healthcare setting. Patient Educ Couns.

[32] Kumar D, Sanders L, Perrin EM, Lokker N, Patterson B, Gunn V, Finkle JP, Franco V, Choi L, Rothman RL (2010). Parental understanding of infant health information: health literacy, numeracy, and the Parental Health Literacy Activities Test (PHLAT). Acad Pediatr.

[33] Yin HS, Sanders LM, Rothman RL, Mendelsohn AL, Dreyer BP, White RO, Finkle JP, Prendes S, Perrin EM (2011). Assessment of health literacy and numeracy among Spanish-speaking parents of young children: Validation of the Spanish Parental Health Literacy Activities Test (PHLAT-Spanish). Acad Pediatr.

[34] U.S. Department of Health and Human Services Food Drug Administration (2009). Guidance for Industry: Patient-Reported Outcome Measures—Use in Medical Product Development to Support Labeling Claims. Health Qual Life Outcomes. vol. 5.

[35] Streiner DL, Norman GR (2008). Health Measurement Scales: A Practical Guide to Their Development and Use.

[36] Buchbinder R, Batterham R, Elsworth G, Dionne CE, Irvin E, Osborne RH (2011). A validity-driven approach to the understanding of the personal and societal burden of low back pain: development of a conceptual and measurement model. Arthritis Res Ther.

[37] Hox JJ, Lyberg LE, Biemer P, Collins M, De Leeuw ED, Dippo C, Schwarz N, Trewin D (1997). From Theoretical Concept to Survey Question. Survey Management and Process Quality.

[38] Patrick DL, Burke LB, Gwaltney CJ, Leidy NK, Martin ML, Molsen E, Ring L (2011). Content validity - Establishing and reporting the evidence in newly developed patient-reported outcomes (PRO) instruments for medical product evaluation: ISPOR PRO good research practices task force report: Part 1 - Eliciting concepts for a new PRO instrument. Value Health.

[39] Flesch R (1948). A new readability yardstick. J Appl Psychol.

[40] Kincaid JP, Fishburne RP, Rogers RL, Chissom BS (1975). Derivation of New readability Formulas (Automated Readability Index, Fog Count and Flesch Reading Ease Formula) for Navy Enlisted Personnel. DTIC Document.

[41] Anderson LW, Krathwohl DR (2001). A Taxonomy for Learning, Teaching, and Assessing: A Revision of Bloom’s Taxonomy of Educational Objectives.

[42] Krathwohl DR (2002). A revision of Bloom’s taxonomy: an overview. Theory Pract.

[43] Leung AYM, Cheung MKT, Lou VWQ, Chan FHW, Ho CKY, Do TL, Chan SSC, Chi I (2013). Development and validation of the Chinese Health Literacy Scale for chronic care. J Health Commun.

[44] Leung AYM, Lou VWQ, Cheung MKT, Chan SSC, Chi I (2012). Development and validation of Chinese Health Literacy Scale for Diabetes. J Clin Nurs.

[45] DeVellis RF (2011). Scale Development: Theory and Applications, Vol. 3.

[46] Lynn MR (1986). Determination and quantification of content validity. Nurs Res.

[47] Willis GB (2005). Cognitive Interviewing: A Tool for Improving Questionnaire Design.

[48] Beatty PC, Willis GB (2007). Research synthesis: the practice of cognitive interviewing. Public Opin Q.

[49] Miles MB, Huberman AM, Saldaña J (2013). Qualitative Data Analysis: A Methods Sourcebook, Vol. 3.

[50] McCarthy B (2011). Family members of patients with cancer: what they know, how they know and what they want to know. Eur J Oncol Nurs.

[51] Polit DF, Beck CT, Owen SV (2007). Is the CVI an acceptable indicator of content validity? Appraisal and recommendations. Res Nurs Health.

[52] Sen A (2002). Health: perception versus observation: self reported morbidity has severe limitations and can be extremely misleading. BMJ.

